# Genetic diversity assessment of sesame core collection in China by phenotype and molecular markers and extraction of a mini-core collection

**DOI:** 10.1186/1471-2156-13-102

**Published:** 2012-11-15

**Authors:** Yanxin Zhang, Xiurong Zhang, Zhuo Che, Linhai Wang, Wenliang Wei, Donghua Li

**Affiliations:** 1 Oil Crops Research Institute of the Chinese Academy of Agricultural Sciences, Key Laboratory of Biology and Genetic Improvement of Oil Crops, Ministry of Agriculture, P.R.China, No.2 Xudong 2nd Rd, Wuhan 430062, China; 2 Gansu Provincial Seed Management Bureau, Lanzhou, 730020, China

**Keywords:** Genetic diversity, Assessment, Sesame, Core collection, Mini-core collection

## Abstract

**Background:**

Sesame (*Sesamum indicum* L.) is one of the four major oil crops in China. A sesame core collection (CC) was established in China in 2000, but no complete study on its genetic diversity has been carried out at either the phenotypic or molecular level. To provide technical guidance, a theoretical basis for further collection, effective protection, reasonable application, and a complete analysis of sesame genetic resources, a genetic diversity assessment of the sesame CC in China was conducted using phenotypic and molecular data and by extracting a sesame mini-core collection (MC).

**Results:**

Results from a genetic diversity assessment of sesame CC in China were significantly inconsistent at the phenotypic and molecular levels. A Mantel test revealed the insignificant correlation between phenotype and molecular marker information (*r* = 0.0043, *t* = 0.1320, *P* = 0.5525). The Shannon-Weaver diversity index (I) and Nei genetic diversity index (h) were higher (I = 0.9537, h = 0.5490) when calculated using phenotypic data from the CC than when using molecular data (I = 0.3467, h = 0.2218). A mini-core collection (MC) containing 184 accessions was extracted based on both phenotypic and molecular data, with a low mean difference percentage (MD, 1.64%), low variance difference percentage (VD, 22.58%), large variable rate of coefficient of variance (VR, 114.86%), and large coincidence rate of range (CR, 95.76%). For molecular data, the diversity indices and the polymorphism information content (PIC) for the MC were significantly higher than for the CC. Compared to an alternative random sampling strategy, the advantages of capturing genetic diversity and validation by extracting a MC using an advanced maximization strategy were proven.

**Conclusions:**

This study provides a comprehensive characterization of the phenotypic and molecular genetic diversities of the sesame CC in China. A MC was extracted using both phenotypic and molecular data. Low MD% and VD%, and large VR% and CR% suggested that the MC provides a good representation of the genetic diversity of the original CC. The MC was more genetically diverse with higher diversity indices and a higher PIC value than the CC. A MC may aid in reasonably and efficiently selecting materials for sesame breeding and for genotypic biological studies, and may also be used as a population for association mapping in sesame.

## Background

Sesame (*Sesamum indicum* L.) has been cultivated in Asia for over 5000 years. In China, sesame is one of the four major oil crops, along with rapeseed, soybean, and peanut. On average (from 2001 to 2010), over 627,000 hectares of sesame are harvested annually, producing over 663,000 tons of sesame seeds, representing about 20% of the world’s production [1]. Furthermore, China has been identified as one of the five sesame diversity centers in classical studies [2,3]. As of 2012, the national gene bank of China has collected, reproduced, and preserved 5550 accessions of sesame.

Abundant plant germplasm resources provide a broad genetic foundation for plant breeding and genetic research. However, large germplasm resources are also difficult to preserve, evaluate, and use [4]. Establishing a core collection (CC) is a favored approach for the efficient exploration and utilization of novel variation in genetic resources [5,6]. The concept of a CC was first proposed by Frankel [7] and later developed by Brown [8]. It involves the selection of a subset from the whole germplasm by certain methods in order to capture the maximum genetic diversity of the whole collection while minimizing accessions and redundancy. To date, CC have been established for many plant species around the world, including peanut [9,10], barley [11], ryegrass [12], soybean [13,14], safflower [15], rice [6,16], olive [17,18], *Brassica rapa*[19], *Cornus officinalis*[20], *Arabidopsis thaliana*[21], *Medicago truncatula*[22], and *Vitis vinifera*[23]. To increase the usefulness of CC, genetic information must be clearly identified and documented [24].

To further reduce the duplication of some accessions in a CC, a ‘mini-core collection’ (MC) can serve as a small, representative subset of the CC. MC have been developed and evaluated for chickpea [25], peanut [26,27], pigeon pea [28], maize [29], sorghum [30], rice [6,31,32], and other crops, promoting the utilization of genetic resources for these plants. For example, Upadhyaya [33] investigated the variability in drought resistance-related traits in the 184 entries of a MC for peanut. The results suggested certain accessions that can be used in peanut improvement programs to develop cultivars with a broad genetic base. Chamberlin et al. [34] evaluated a U.S. peanut MC using a molecular marker for resistance to *Sclerotinia minor* Jagger. They identified 39 accessions as new potential sources for resistance and targets for further evaluation. Using association analysis, Li et al. [35] mapped quantitative trait loci (QTLs) for improving grain yield using the USDA rice MC. Wang et al. [36] conducted association analysis of seed quality traits in a U.S. peanut (*Arachis hypogaea* L.) MC. In addition, Sharma et al. [37] identified new sources of resistance to Fusarium wilt and sterility mosaic disease using a pigeon pea MC and found that the diverse accessions with resistance would be useful in pigeon pea resistance breeding programs.

India, China, and Korea are the world’s leading countries for sesame germplasm collection and preservation, as well as research on sesame CC establishment. Bisht et al. [38] investigated 19 phenotypic and agronomic traits in 3129 sesame accessions from seven eco-geographical regions in India and established a sesame CC consisting of 362 accessions in India. Kang et al. [39] investigated 12 agronomic traits in 2246 sesame accessions from ten agro-climate zones preserved in the Rural Development Administration (RDA) Genebank in Korea and established a sesame CC of 475 accessions. In China, a systematic study of technical methods for the establishment of a sesame CC was conducted in cooperation with the International Plant Genetic Resources Institute (IPGRI). A sesame CC containing 453 accessions was established from the basic collection (BC) of 4251 accessions collected in China and 15 other countries using Ward’s clustering method and a stratified sampling strategy based on data for 14 phenotypic traits [40]. The major objective for establishing sesame germplasm CC in China, Korea, and India has been to utilize germplasm more effectively. However, up until now, no comprehensive study of sesame CC genetic diversity has been carried out at either a phenotypic level or molecular level.

This study examined the genetic diversity of accessions from the sesame CC in China. The objectives of this investigation were to comprehensively explore the characteristics of genetic diversity at both a phenotypic level and a molecular level, and then to provide a theoretical foundation for effectively protecting and utilizing sesame genetic resources. Furthermore, a MC was extracted using an advanced maximization strategy based on both phenotypic and molecular data with the aim of promoting reasonable and efficient applications of sesame accessions in breeding and genotypic biological studies.

## Results

### Polymorphism detected by molecular markers

A total of 36 sequence-related amplified polymorphism (SRAP) primer combinations and ten simple sequence repeat (SSR) primer pairs were employed randomly to screen polymorphism between 12 accessions (typical accessions from each group). Of these, 11 SRAP primer combinations and 3 SSR primer pairs amplified abundant, clear, and repeatable fragments. They were then employed to evaluate the genotypic diversity of 453 accessions in the CC. In total, 175 amplified fragments were detected and 126 of them were polymorphic, with a polymorphism rate of 72%. The number of fragments detected by each primer ranged from 2 (GBssr-sa-173) to 26 (Me07Em06), with an average of 12.5. The number of polymorphic fragments detected by each primer ranged from 2 (GBssr-sa-173) to 17 (Me08Em05), with an average of 9.

### Genetic diversity analysis of the CC

The Euclidean genetic distance (GD) was calculated based on data for standardized values of 14 phenotype traits. The Jaccard genetic similarity coefficient (GS) was evaluated based on data for SRAP and EST-SSR markers for each of the nine groups in the sesame CC (Table 1). Of the nine groups, the first seven were divided according to the seven agro-ecological zones in China [40]. Group VIII was a group of cultivars released in China, and group IX was a group of exotic accessions. Because of the different accession types and origins, some groups in the sesame CC are divergent and others are not. The GD between accessions was ranged from 1.4883 to 10.9347 (with an average of 5.0854) in the nine groups. The average GD (5.2158) in group VIII was the highest, and the average GD (5.0702) in group IX was the lowest, indicating that group IX had the closest genetic relationship between accessions, while group VIII had the farthest genetic relationship, as evaluated by phenotypic data.

**Table 1:** Euclidean genetic distance and Jaccard similarity coefficient for each group

**Group**	**Euclidean genetic distance based on phenotypic data**			**Jaccard similarity coefficient based on molecular data**		
	**Max**	**Min**	**Aver**	**Max**	**Min**	**Aver**
I	9.1093	2.0387	5.1530	0.9892	0.4426	0.7447
II	8.9414	1.4883	5.1728	0.9765	0.4370	0.7339
III	8.1180	2.3699	5.1929	0.9697	0.5405	0.7865
IV	8.5937	2.2524	5.1956	0.9789	0.4344	0.7214
V	8.5662	2.2726	5.2057	0.9892	0.4000	0.7030
VI	9.0900	2.2553	5.1893	0.9789	0.4359	0.7208
VII	8.3926	2.5218	5.1979	0.9592	0.4711	0.6857
VIII	7.4283	2.8483	5.2158	0.9560	0.5776	0.7486
IX	10.9347	2.4313	5.0702	0.9688	0.5000	0.6999

The pairwise GS between all 453 of the accessions in the nine groups ranged from 0.4000 to 0.9892, with an average of 0.7060. The average GS (0.7865) was the highest for group III, followed by that for group VIII (0.7486). The lowest average GS was found for group VII (0.6857), indicating that genetic relationships were the closest between accessions for group III, and the second closest for group VIII. Accessions in group VII had the most distant genetic relationship, as evaluated by molecular data.

**Figure 1:**
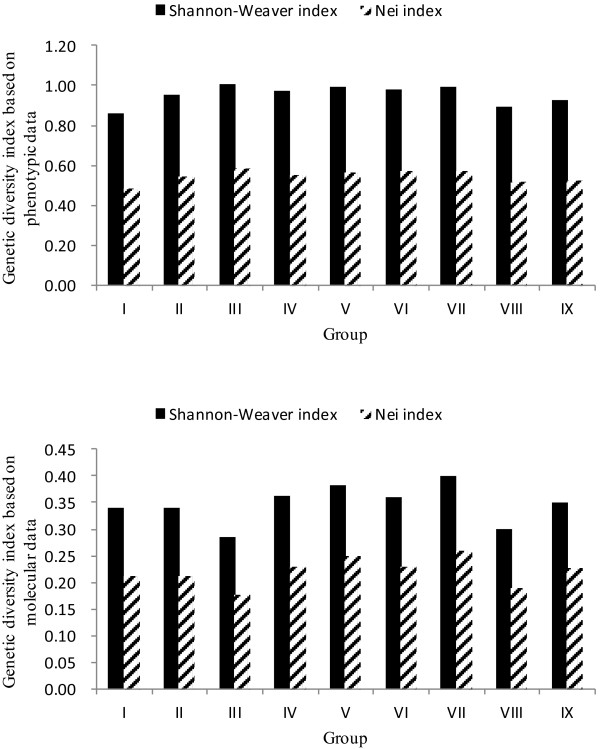
Histogram of genetic diversity indices based on phenotypic and molecular data for nine groups in the core collection (CC).

The most widely used biodiversity indices, the Shannon-Weaver diversity index (I) and the Nei genetic diversity index (h), were calculated for the nine groups based on phenotypic and molecular data (Figure 1). Evaluated using phenotypic data, the Shannon-Weaver diversity index among the nine groups ranged from 0.8613 to 1.0087, with an average of 0.9537. The Nei genetic diversity index ranged from 0.4892 to 0.5865, with an average of 0.5490. However, both of these indices were much lower when evaluated using molecular data. Molecularly, the Shannon-Weaver diversity index ranged from 0.2859 to 0.3987, with an average of 0.3467, and the Nei genetic diversity index ranged from 0.1773 to 0.2615, with an average of 0.2218. Conversely, using phenotypic data, the maximum diversity indices values (I = 1.0087, h = 0.5865) among the accessions in group III indicated that they were genetically more diverse than other groups, whereas the genetic diversity of accessions in group I was limited (I = 0.8613, h = 0.4892). The genetic diversity of the nine groups as evaluated using molecular data was significantly different from the genetic diversity evaluated using phenotypic data. The maximum diversity indices values (I = 0.3987, h = 0.2615) for group VII indicated that they were genetically more diverse than in other groups, whereas genetic diversity was weak in group III (I = 0.2859, h = 0.1773). This trend in the diversity variation is consistent with that of the pair-wise GS discussed above.

### Principal coordinate (PCO) analysis of the CC

**Figure 2:**
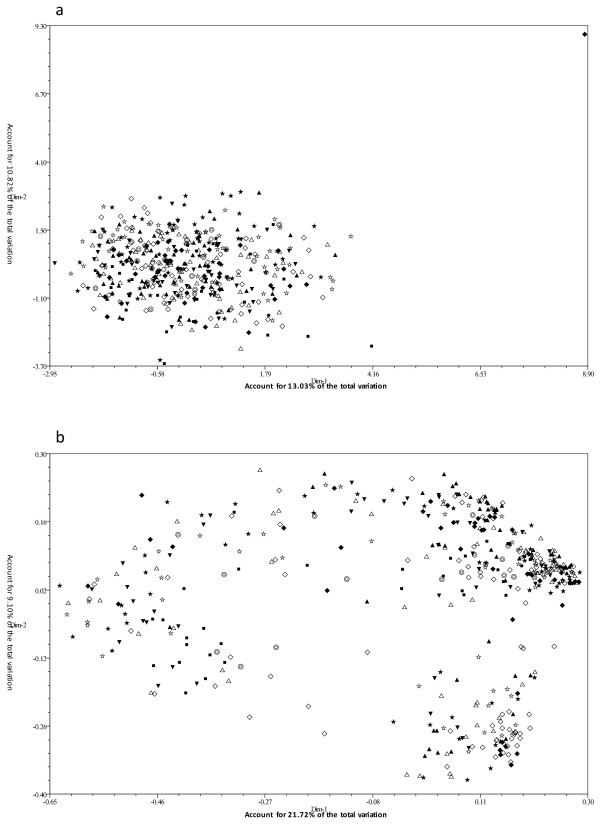
Scatter plot of principal coordinates analysis for the 453 accessions in the CC.

Results of the principal coordinate (PCO) analysis based on phenotypic and molecular data are shown in scatter plots of the first two principal components, respectively (Figures 2a and 2b). The first and second principal components respectively explained 13.03% and 10.82% of the variance in the phenotypic data (Figure 2a) and 21.72% and 9.10% of the variance in the molecular data (Figure 2b). There were significant differences between the distributions of the accessions in the two figures; however, no significance was found for the distributions of each group in Figures 2a and 2b. Accessions from groups I to IX were highly concentrated in Figure 2a, whereas the distribution of accessions was comparatively disperse in Figure 2b, which indicates that genetic relationships between accessions evaluated using phenotypic and molecular data were significantly discrepant. To obtain further evidence, the relationship between the phenotypic-based clustering matrix and the molecular-based clustering matrix was tested with a Mantel test. The results revealed that the goodness of fit between phenotype and molecular marker analyses was not significant (*r* = 0.0043, *t* = 0.1320, *P* = 0.5525) in detecting genetic relationship of the accessions.

Scatter plot based on (a) phenotypic data and (b) molecular data. Accessions from groups I, II, III, IV, V, VI, VII, VIII, and IX are marked with ♦, ⋄, ▴, △, ★, ☆, ▾, ⊚, and ▪, respectively.

### MC extraction and comparison with CC

On the basis of data of 14 phenotypic traits and 126 polymorphic markers, a heuristic search applying an advanced maximization strategy identified a MC of 184 accessions from the 453 accessions in the CC, with frequencies of 40.62% for the CC and 4.33% for the BC. Table 2 lists the similarity of distribution frequencies between the MC and CC for each of the nine groups, tested using *χ2* with one degree of freedom. Except for groups VIII and IX, the other seven groups had nonsignificant *χ2* values ranging from 0.003 to 1.377, with a probability (*P*) from 0.241 to 0.959, which showed a homogeneous distribution between the MC and CC in these groups.

**Table 2:** Distribution frequency comparison and representation analysis of accessions from the CC and MC among the nine groups

**Group**	**CC**		**MC**		***χ2***	***P***	**MD%**	**VD%**	**VR%**	**CR%**
	**Number**	**%**	**Number**	**%**						
I	42	9.27	18	9.78	0.228	0.633	0.64	29.66	119.12	96.08
II	78	17.22	28	15.22	0.534	0.465	1.71	27.93	121.82	95.75
III	69	15.23	21	11.41	1.377	0.241	0.93	16.18	110.35	95.57
IV	56	12.36	23	12.50	0.003	0.959	2.55	18.87	110.03	92.54
V	55	12.14	22	11.96	0.022	0.883	1.12	22.3	115.01	96.19
VI	57	12.58	22	11.96	0.022	0.883	2.84	25.5	117.77	97.64
VII	47	10.38	16	8.70	0.244	0.621	2.29	25.97	118.56	92.76
VIII	18	3.97	14	7.61	4.862	0.027	0.82	11.79	106.5	95.32
IX	31	6.84	20	10.87	5.234	0.022	1.84	24.98	114.54	100
Total	453	100.00	184	100.00	/	/	/	/	/	/
Mean	/	/	/	/	/	/	1.64	22.58	114.86	95.76

*χ2 *and *P *indicate *χ2 *values with one degree of freedom and the corresponding probability, respectively MD denotes the mean difference percentage, VD represents the variance difference percentage, VR indicates the variable rate of the coefficient of variance, and CR is the coincidence rate of range.

**Figure 3:**
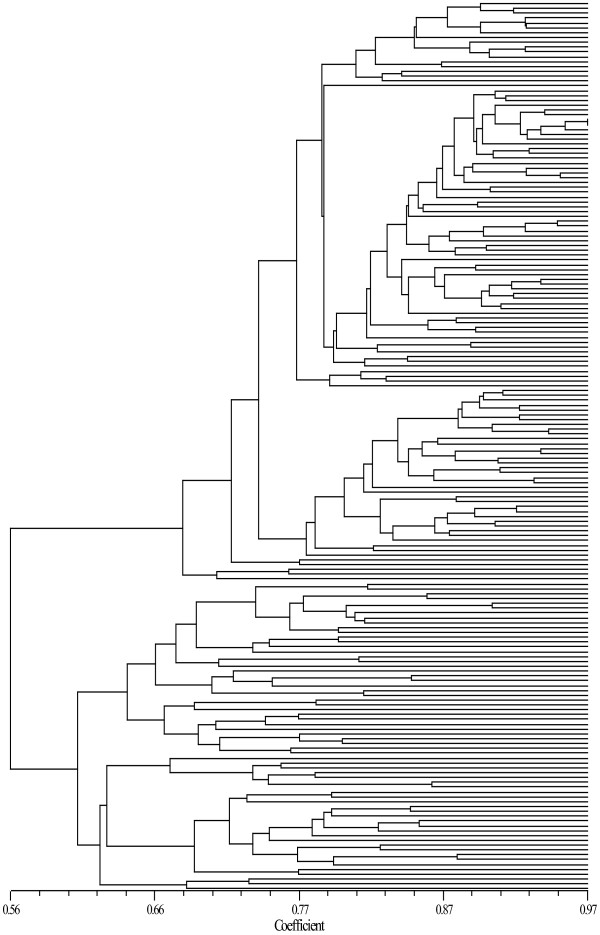
UPGMA dendrogram of the 184 accessions in the sesame MC based on molecular data.

Phylogenetic analysis of the CC (Additional file 1: figure S1) and MC (Figure 3) accessions was performed based on molecular data. The unweighted pair group method with arithmetic mean (UPGMA) dendrogram in Additional file 1: Figure S1 suggests the balanced distribution of MC accessions within CC accessions. A pairwise genetic similarity (GS) coefficient between accessions in the MC ranged from 0.4000 to 0.9681, with an average of 0.6615, which was smaller than the average GS coefficient of 0.7060 in the CC, indicating a higher genetic diversity in the MC.

The mean difference percentage (MD, %), coincidence rate of range (CR, %), variance difference percentage (VD, %), and variable rate of coefficient of variance (VR, %) are used to comparably evaluate the properties of MC with CC. Over all the 14 phenotypic traits, the MD% between the CC and MC ranged from 0.64% to 2.84% in the nine groups (Table 2), with an average of 1.64%, far less than the significance level of 20% [41]. The VD% ranged from 11.79% to 29.66%, with an average of 22.58%, slightly higher than the significance level of 20% [41]. The VR% compares the coefficient of variation values for the phenotypic traits measured in the CC, with a representative general subset, and determines how well the variance is being represented in the representative subset [31]. More than 100% of VR% is required for a subset to be representative of its original CC [41]. The resulting average VR% of the MC was 114.86% (with a range of 106.50% to 121.82%), indicating that good representation of the original CC was achieved. The CR% indicates whether the distribution ranges of each trait in the MC are well represented when compared to the CC. The resulting average CR% in the nine groups was 95.76% (with a range of 92.54% to 100%), indicating homogeneous distribution ranges for the phenotypic traits, because CR was greater than 80% [42].

**Figure 4:**
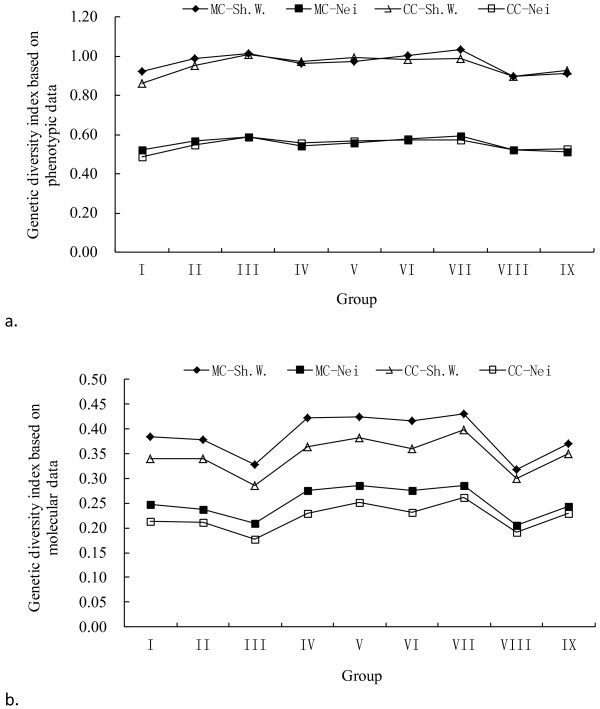
**Genetic diversity indices of accessions in the MC and CC among the nine groups based on phenotypic and molecular data. ‘**Sh.W.’ and ‘Nei’ indicate the Shannon-Weaver diversity index and Nei genetic diversity index, respectively.

The Shannon-Weaver diversity index and the Nei genetic diversity index of accessions from each of the nine groups in MC were calculated based on phenotypic and molecular data and compared with CC (Table 3, Figure 4). The Shannon-Weaver diversity index, among the nine groups of MC based on phenotypic data, ranged from 0.8977 to 1.0329, with an average of 0.9677. The Nei genetic diversity index ranged from 0.5110 to 0.5938, with an average of 0.5530. When based on molecular data, the Shannon-Weaver diversity index of MC ranged from 0.3187 to 0.4310, with an average of 0.3861, and the Nei genetic diversity index ranged from 0.2062 to 0.2863, with an average of 0.2519. Diversity indices evaluated using phenotypic data among each group were higher than when evaluated using molecular data. Distribution trends for both of the diversity indices based on phenotypic and molecular data were very similar between MC and CC among the nine groups. Results of pairwise *t*-tests indicated that neither diversity index differed significantly between the CC and MC based on phenotypic data, but that they were significantly (*P*<0.0001) higher in the MC than in the CC based on molecular data.

**Table 3:** Genetic diversity indices and PIC values among the nine groups in the MC and CC

**Group**	**Genetic diversity index based on phenotypic data**				**Genetic diversity index based on molecular data**				**PIC value from molecular data**	
	**MC-Sh.W.**	**CC-Sh.W.**	**MC-Nei**	**CC-Nei**	**MC-Sh.W.**	**CC-Sh.W.**	**MC-Nei**	**CC-Nei**	**MC**	**CC**
I	0.9211	0.8613	0.5205	0.4892	0.3847	0.3398	0.2471	0.2138	0.2033	0.1780
II	0.9897	0.9532	0.5662	0.5476	0.3783	0.3408	0.2380	0.2119	0.1973	0.1767
III	1.0137	1.0087	0.5860	0.5865	0.3287	0.2859	0.2090	0.1773	0.1721	0.1482
IV	0.9651	0.9742	0.5424	0.5558	0.4228	0.3635	0.2766	0.2295	0.2226	0.1900
V	0.9730	0.9929	0.5577	0.5662	0.4248	0.3830	0.2852	0.2511	0.2262	0.2029
VI	1.0038	0.9807	0.5768	0.5715	0.4156	0.3596	0.2755	0.2315	0.2203	0.1891
VII	1.0329	0.9905	0.5938	0.5753	0.4310	0.3987	0.2863	0.2615	0.2289	0.2111
VIII	0.8977	0.8966	0.5223	0.5227	0.3187	0.2993	0.2062	0.1909	0.1668	0.1558
IX	0.9123	0.9249	0.5110	0.5266	0.3699	0.3497	0.2429	0.2286	0.1954	0.1841
*t* value	1.52		0.73		8.50		7.98		8.58	
*P* value	0.1668		0.4836		<0.0001		<0.0001		<0.0001	

‘Sh.W.’ and ‘Nei’ indicate the Shannon-Weaver diversity index and Nei genetic diversity index, respectively.

The polymorphism information content (PIC) value of each group in the MC ranged between 0.1668 and 0.2289, with an average of 0.2037, whereas PIC ranged from 0.1482 to 0.2111 in the CC, with an average of 0.1818. Results of pairwise *t*-tests indicated that the PIC value in the MC was significantly (*P*<0.0001) higher than that in the CC (Table 3). In addition to the genetic diversity indices and the PIC values, we also compared the loci and alleles of molecular markers between the MC and the CC. The allele frequency was diverse, from 0.44% to 100% in the CC, with an average of 50.78%, whereas it ranged between 1.09% and 100% in the MC, with an average of 49.98%. No allele was missing, and 100% of the allelic diversity at the tested loci was represented in the MC.

### Validation of the sampling strategy for extracting the MC

To validate the sampling strategy (the advanced maximization strategy) used for extracting the MC, an alternative strategy of random sampling was also applied, and a second MC composed of 184 accessions was established and investigated. Using phenotypic data, the Shannon-Weaver diversity index of each group from the second MC ranged from 0.7749 to 0.9784, with an average of 0.9020, and the Nei genetic diversity index ranged from 0.4652 to 0.5767, with an average of 0.5303. When based on molecular data, the Shannon-Weaver diversity index ranged from 0.2498 to 0.3609, with an average of 0.3201, and the Nei genetic diversity index ranged from 0.1610 to 0.2334, with an average of 0.2054. Results of a pairwise *t*-tests indicated that the diversity indices of the second MC were significantly lower than those of the first MC (Table 4), which proved to be an advantage in capturing genetic diversity and validation and in extracting the MC using an advanced maximization strategy.

**Table 4:** **Genetic diversity indices of accessions in MC extraction using two different strategies and a significance of difference analysis based on *t*-tests**

**Group**	**Genetic diversity index based on phenotypic data**				**Genetic diversity index based on molecular data**			
	**RM-Sh.W.**	**M-Sh.W.**	**RM-Nei**	**M-Nei**	**RM-Sh.W.**	**M-Sh.W.**	**RM-Nei**	**M-Nei**
I	0.8458	0.9211	0.4908	0.5205	0.3291	0.3847	0.2099	0.2471
II	0.9241	0.9897	0.5401	0.5662	0.3173	0.3783	0.1958	0.2380
III	0.9784	1.0137	0.5755	0.5860	0.2568	0.3287	0.1610	0.2090
IV	0.9374	0.9651	0.5378	0.5424	0.3454	0.4228	0.2171	0.2766
V	0.9428	0.9730	0.5419	0.5577	0.3583	0.4248	0.2314	0.2852
VI	0.9047	1.0038	0.5553	0.5768	0.3181	0.4156	0.2057	0.2755
VII	0.9614	1.0329	0.5767	0.5938	0.3609	0.4310	0.2334	0.2863
VIII	0.7749	0.8977	0.4652	0.5223	0.2498	0.3187	0.1655	0.2062
IX	0.8487	0.9123	0.4897	0.5110	0.3453	0.3699	0.2285	0.2429
*t* value	−6.18		−4.52		−10.17		−8.87	
*P* value	0.0003		0.0019		<0.0001		<0.0001	

‘RM-Sh.W.’ and ‘M-Sh.W.’ indicate the Shannon-Weaver diversity index of the MC extracted using the random sampling strategy and the advanced maximization strategy, respectively. ‘RM-Nei’ and ‘M-Nei’ indicate the Nei genetic diversity index of MC extracted by the random sampling strategy and the advanced maximization strategy, respectively.

## Discussion

### Significance of the phenotypic and molecular genetic diversities of the sesame CC

The sesame CC in China is one of only three sesame CC in the world and differs from the other two collections [41,42] in that it was developed from a basic collection (BC) with a larger quantity (4251 accessions), broader origin (16 countries), more diverse types (landrace, cultivar, special material), and more genetic diversity. Since sesame CC in China was established in 2000 [40], no study has examined its genetic diversity either at a phenotypic or molecular level. The present study comprehensively characterized the phenotypic and molecular genetic diversities of this CC. Our results will provide both technical guidance and a theoretical basis not only for further collection of sesame germplasm (from areas or agro-ecological zones with higher diversity) and the effective protection of sesame accessions (with rare alleles), but also for reasonable application and comprehensive analysis of sesame genetic resources. Information from a combined phenotypic and molecular genetic analysis of the CC can also be used to design parental crosses that maximize genetic polymorphisms for important traits.

### Genetic diversity assessments of phenotypic and molecular data were significantly inconsistent

This study assessed genetic diversity in the nine groups of sesame CC in China both at the phenotypic and molecular levels, but the results were inconsistent. Evaluated based on phenotypic data, the Euclidean genetic distance indicated that genetic relationships were the closest between accessions in group IX and the most distant in group VIII. The Shannon-Weaver and Nei genetic diversity indices indicated that accessions in group III were genetically more diverse than in other groups, while group I displayed the least genetic diversity. Evaluated using molecular data, the Jaccard genetic similarity coefficient and genetic diversity indices indicated that the genetic relationships were nearest between accessions in group III, followed by accessions in group VIII. Accessions in group VII had the most distant genetic relationship. Furthermore, the phenotypic-based cluster did not correspond with the molecular-based cluster; the correlation coefficient (*r* = 0.0043) for the two clustering matrices tested by a Mantel test showed an insignificant correlation between phenotype and molecular marker information. This result is much lower than that found in safflower (*r* = 0.12) by Johnson et al. [43] and also much lower than that found by Reed and Frankham [44] in 71 datasets (*r* = 0.217). Reed and Frankham [44] suggested that molecular measures of genetic diversity have a very limited ability to predict quantitative genetic variability. Therefore, the combination of phenotypic-based and molecular-based analyses in genetic diversity assessments of the sesame CC is very important.

### Why were genetic diversity indices of the CC much higher when evaluated using phenotypic data?

Both the Shannon-Weaver diversity index and the Nei genetic diversity index were much higher (I = 0.9537, h = 0.5490) when calculated using phenotypic data in the CC than when using molecular data (I = 0.3467, h = 0.2218). The sesame CC used in this study was extracted from the BC based on phenotypic traits data. Molecular data were not referred to because of the lack of molecular marker techniques; therefore genetic diversity was comparatively higher when examined on a phenotypic level. Genetic diversity on a molecular level was not considered in the establishing the CC. The high genotypic coefficient of similarity and the small phenotypic coefficient of distance between some accessions indicated that there were duplicates or near duplicates included within the CC. Therefore, in this study, extraction of a MC from the CC was performed based on both phenotypic and molecular data, using a diversity maximization strategy. Some genetically similar accessions were removed from the CC by further reduction; thus the genetic diversity indices evaluated by molecular data in the MC (I = 0.3861, h =0.2519) were significantly higher than in the CC, and the genetic diversity indices evaluated by phenotypic data (I = 0.9677, h =0.5530) were also enhanced.

### Sampling strategy for the MC extraction and representation

Using a heuristic algorithm, Kim et al. [42] developed the PowerCore program, which selects a subset of accessions with a higher diversity representing the total coverage of marker alleles and trait states present in the entire collection, applying an advanced maximization strategy. Zhao et al. [45] selected 50 rice cultivars from each of Korea, China, and Japan from the RDA Genebank using the Powercore program and analyzed their genetic diversity and population structure using SSR Markers. Belaj et al. [18] developed a CC of olive (*Olea europaea* L.) based on molecular markers and agronomic traits, using the PowerCore and MSTRAT programs [46]. Their results suggested that the CC extracted by PowerCore may be of special interest for genetic conservation applications in olive, owing to PowerCore’s high efficiency at capturing all of the allele/trait states found in the entire collection. Subsequent applications of PowerCore also suggested its effectiveness in establishing a CC that retains all characteristics of qualitative traits and all classes of quantitative ones [6].

In this study, PowerCore was used to extract a sesame MC containing 184 accessions from the CC and the resulting collection was compared to that obtained by random sampling. The advantage of the advanced maximization strategy of PowerCore in capturing genetic diversity and in validating MC extraction was illustrated. An MC with low MD% and VD% and large VR% and CR% can be considered to provide a good representation of the genetic diversity in the initial CC [41,42]. The similarity of diversity index distributions between the MC and CC among the nine groups also showed that the selected MC provides a sound description of the sesame genetic diversity found in China. In addition, the *χ2* test results showed homogeneous distribution frequencies between the MC and CC from group I to group VII, with two exceptions (groups VIII and IX). The distribution frequencies for Group VIII were 3.97% in the CC and 7.61% in the MC, whereas those for group IX were 6.84% in the CC and 10.87% in the MC. The distribution frequencies of these two groups from the MC were significantly higher than from the CC, which may be attributed to their special accession types. Accessions from group VIII were made up of 18 cultivars released in China, and accessions from group IX were 31 exotic landraces from 15 countries. The accession numbers were both very limited, and to maintain the genetic diversity of these two groups, it was necessary to increase their frequencies in the MC.

### Limitations of this study and future development of the sesame MC

This study established a MC, but continued research on sesame germplasm is necessary. More accessions are being added to the BC, and with these accessions must also be updated in the CC and MC in a dynamic manner. The sesame MC presented here will be useful for efficiently selecting accessions with maximum diversity for sesame breeding, for selecting parents to generate mapping populations, or for further evaluation and restructuring of the CC. Furthermore, the MC, with its smaller number of accessions than the CC, can aid in comprehensive investigations of important traits and molecular markers in sesame. The combination of phenotype and molecular marker data can be used directly for association mapping and for developing key molecular markers associated with important traits.

## Conclusion

This study presented a comprehensive characterization of the phenotypic and molecular genetic diversities of sesame CC in China. We extracted an MC containing 184 accessions from the CC based on both of phenotypic and molecular data. Low MD% and VD% and large VR% and CR% suggested that the MC provided a good representation of the genetic diversity of the original CC; it was more genetically diverse with higher diversity indices and a higher PIC value than the CC. The development of a MC may aid in reasonably and efficiently selecting materials for sesame breeding and for genotypic biological studies, and may also be used as a population for association mapping in sesame.

## Materials and methods

### Plant materials

All of the 453 accessions from the sesame germplasm CC in China were used in this study. Among them, 404 indigenous landraces were from seven agro-ecological zones (29 provinces) in China, which were divided according to climatic and geographic characteristics and the planting system. There were also 18 released cultivars from China and 31 exotic accessions from 15 other countries around the world. The accessions were divided into nine groups (with group codes I to IX), in which the first seven groups covered the seven agro-ecological zones in China [40]. Group VIII was the group of cultivars released in China, and group IX was the group of exotic accessions (Table 5).

**Table 5:** Description of the 453 sesame accessions used in this study

**Group**	**Acc. No.**	**Type**	**Origin**	**Annotation**
I	42	Landrace	Northeast and northwest of China	agro-ecological zone I
II	78	Landrace	Northern China	agro-ecological zone II
III	55	Landrace	The Yellow River and Huai River valley	agro-ecological zone III
IV	69	Landrace	The Yangtze and Han River valley	agro-ecological zone IV
V	56	Landrace	Middle and lower of Yangtze valley	agro-ecological zone V
VI	57	Landrace	South-central of China and southern China	agro-ecological zone VI
VII	47	Landrace	Southwest of China	agro-ecological zone VII
VIII	18	Cultivar released	China	-
IX	31	Landrace	Exotic accessions	-

### Phenotype data mining and analysis

The following 14 genetically stable and important agronomical traits were investigated: growth period, plant type, number of locules, flower number per nod, stem hairiness, flower color, seed color, capsule dehiscence, tolerance of water-logging, resistance to stem spot wilt, resistance to wilt, 1000-seed weight, oil content, and protein content. Values of these 14 traits recorded in a database [40] were used to conduct the following statistical analysis. Quantification of qualitative traits followed the method of Zhang et al. [40]. Phenotype data were standardized first using the standardization program of NTSYS-pc software version 2.1 [47]. Genetic distance (GD) was calculated using the Interval data program and the EUCLID (Euclidean) coefficient, which is likely the most commonly used type of distance. Principal coordinate (PCO) analysis was undertaken using principal component analysis programs such as DCENTER and EIGEN based on genetic distance matrices, and a scatter plot was generated. Additionally, the Shannon-Weaver diversity index and the Nei genetic diversity index were estimated using POPGENE version 1.32 [48].

### DNA extraction, PCR amplification and electrophoresis

The total genomic DNA of 453 accessions was prepared from young healthy leaves according to the cetyltrimethylammonium bromide (CTAB) method [49] with some modification of the components of the CTAB buffer (8.18 g sodium chloride and 2 g CTAB in a total volume of 100 ml of 20 mM EDTA, 100 mM Tris, pH set to 8.0). A total of 36 SRAP primer combinations (between 11 Me and 11 Em primers) and 10 SSR primer pairs were used (Table 6) to scan the polymorphism. The sequences of SRAP and SSR primers were referenced from Li et al. [50] and Dixit et al. [51], respectively. Polymerase chain reaction (PCR) amplification of all SRAP markers was conducted in 20 μL solution containing 80 ng of DNA, 50 ng of forward primers, 50 ng of reverse primers, 1 × buffer (MBI), 4 mmol of Mg2+, 0.40 mmol of dNTPs, and 1 U *Taq* polymerase (MBI). The PCR profile was an initial denaturation at 94°C for 2 min, followed by four cycles of 94°C for 1 min, 35°C for 1 min, and 72°C for 1 min, then 34 cycles of 94°C for 1 min, 50°C for 1 min, and 72°C for 1 min, and then a final incubation at 72°C for 5 min and 4°C thereafter. A PCR of all SSR markers was conducted according to Zhang et al. [52]. All PCRs were conducted in 96-well plates in a PTC-100 thermocycler (MJ Research, Watertown, MA). PCR products were size-separated on 6% denaturing polyacrylamide gels. The electrophoresis parameters and silver staining of gels were based on the protocols of Lin et al. [53].

**Table 6:** Sequences of primers used in this study

**Forward primer code**	**Sequence (5’—3’)**	**Reverse primer code**	**Sequence (5’—3’)**
Me01	TGAGTCCAAACCGGATA	Em01	GACTGCGTACGAATTAAT
Me02	TGAGTCCAAACCGGAGC	Em02	GACTGCGTACGAATTTGC
Me03	TGAGTCCAAACCGGAAT	Em03	GACTGCGTACGAATTGAC
Me04	TGAGTCCAAACCGGACC	Em04	GACTGCGTACGAATTTGA
Me05	TGAGTCCAAACCGGAAG	Em05	GACTGCGTACGAATTAAC
Me06	TGAGTCCAAACCGGTAA	Em06	GACTGCGTACGAATTGCA
Me07	TGAGTCCAAACCGGTCC	Em07	GACTGCGTACGAATTCAA
Me08	TGAGTCCAAACCGGTGC	Em08	GACTGCGTACGAATTCTG
Me09	TGAGTCCAAACCGGACG	Em09	GACTGCGTACGAATTCGA
Me10	TGAGTCCAAACCGGACT	Em10	GACTGCGTACGAATTCAG
Me11	TGAGTCCAAACCGGAGG	Em11	GACTGCGTACGAATTCCA
GBssr-sa-05-F	TCATATATAAAAGGAGCCCAAC	GBssr-sa-05-R	GTCATCGCTTCTCTCTTCTTC
GBssr-sa-08-F	GGAGAAATTTTCAGAGAGAAAAA	GBssr-sa-08-R	ATTGCTCTGCCTACAAATAAAA
Sesame-09-F	CCCAACTCTTCGTCTATCTC	Sesame-09-R	TAGAGGTAATTGTGGGGGA
GBssr-sa-33-F	TTTTCCTGAATGGCATAGTT	GBssr-sa-33-R	GCCCAATTTGTCTATCTCCT
GBssr-sa-72-F	GCAGCAGTTCCGTTCTTG	GBssr-sa-72-R	AGTGCTGAATTTAGTCTGCATAG
GBssr-sa-108-F	CCACTCAAAATTTTCACTAAGAA	GBssr-sa-108-R	TCGTCTTCCTCTCTCCCC
GBssr-sa-123-F	GCAAACACATGCATCCCT	GBssr-sa-123-R	GCCCTGATGATAAAGCCA
GBssr-sa-173-F	TTTCTTCCTCGTTGCTCG	GBssr-sa-173-R	CCTAACCAACCACCCTCC
GBssr-sa-182-F	CCATTGAAAACTGCACACAA	GBssr-sa-182-R	TCCACACACAGAGAGCCC
GBssr-sa-184-F	TCTTGCAATGGGGATCAG	GBssr-sa-184-R	CGAACTATAGATAATCACTTGGAA

### Molecular marker data mining and analysis

All of the major DNA fragments were recorded as either 1 or 0 representing the presence or absence of the band, respectively. The pairwise genetic similarity coefficient (GS) was calculated using Jaccard coefficient [54] by the SIMQUAL program of NTSYS-pc software version 2.1 [47]. Principal coordinate (PCO) analysis was conducted using principal component analysis programs such as DCENTER and EIGEN of the NTSYS-pc based on genetic similarity matrices to generate a scatter plot. The Shannon-Weaver and Nei genetic diversity indices were estimated using POPGENE version 1.32 [48]. The relationship between the phenotypic-based clustering matrix and the genotypic-based clustering matrix was tested using a Mantel test [55,56]. An unweighted pair group method with arithmetic mean (UPGMA) dendrogram was created using MEGA version 4.1 [57]. Polymorphism information content (PIC) was calculated using PowerMarker version 3.25 [58].

### Extracting the MC from the CC

On the basis of the phenotype and molecular marker data, a MC was extracted from the CC using PowerCore software [42], which can represent all the alleles identified by the molecular markers and classes of the phenotypic observations. The software applies an advanced maximization strategy implemented through a modified heuristic algorithm. The resulting MC was compared with the original CC to assess its homogeneity as follows. Chi-squared (*χ2*) tests were used to contrast the similarity of the distribution frequency between the MC and the CC between each group, and homogeneity was further evaluated using the mean difference percentage (MD, %), coincidence rate of range (CR, %), variance difference percentage (VD, %), and variable rate of the coefficient of variance (VR, %) according to Hu et al. [41] and Kim et al. [42]. The Shannon-Weaver diversity index and the Nei diversity index of the MC were estimated using phenotypic data and molecular data, and the significance of genetic diversity index differences between the MC and CC was analyzed using pairwise *t*-tests.

## Competing interests

The authors declare that they have no competing interests.

## Authors’ contributions

YZ designed the study, carried out the molecular marker studies, performed the statistical analysis, and drafted the manuscript. XZ conceived of the study, participated in its design and coordination, provided the phenotypic traits data, and helped to draft the manuscript. ZC conducted the molecular marker studies. LW participated in the statistical analysis and helped to draft the manuscript. WW participated in the statistical analysis. DL participated in the molecular marker studies. All authors read and approved the final manuscript.

## Supplementary Material

Additional file 1: Figure S1 UPGMA dendrogram of the 453 accessions in the sesame CC based on molecular data. The 184 accessions in the MC are indicated by red rhombuses. Click here for file
